# Bridging the Gap: A Phenomenological Study of Transfer Students’ Journey into Professional Nursing

**DOI:** 10.3390/nursrep15020072

**Published:** 2025-02-18

**Authors:** Seungeun Oh, Kyunghwa Lee, Hyungkyun Mok, Kyuhee Jo

**Affiliations:** 1 Department of Nursing, Seoul Women’s College of Nursing, 38, Ganhodae-ro, Seodaemun-gu, Seoul 03617, Republic of Korea; seoh@snjc.ac.kr; 2 Kunsan College of Nursing, 7 Dong Gaejung-gil, Kunsan-si 54068, Republic of Korea; khhong0315@kcn.ac.kr; 3 Department of Health Administration, Hanyang Women’s University, 222, Wangsimni-ro, Seongdong-gu, Seoul 04763, Republic of Korea; 4 College of Nursing, Korea University, Anam-Dong, Seongbuk-Gu, Seoul 02841, Republic of Korea

**Keywords:** nursing transfer students, lived experience, phenomenology, Colaizzi’s method

## Abstract

**Background/Objectives**: South Korea has expanded nursing transfer programs to address its ongoing nursing shortage, but research on transfer students’ experiences remains scarce, and studies on academic persistence and attrition remain limited. This study aimed to explore the lived experiences of nursing transfer students during their first year in nursing school and to emphasize the need for a targeted support system within nursing programs. **Methods:** in-depth interviews with 15 nursing transfer students were analyzed using Colaizzi’s method to identify key adaptation challenges and coping strategies. **Results**: Four key themes emerged: (1) second-chance pursuit under anxiety, (2) navigating ambiguous uncertainty, (3) standing alienated at the community periphery, and (4) reshaping: embracing professional identification. The themes explain transfer students’ challenges when adapting to nursing education. **Conclusions**: This study provides valuable insights into the unique experience of nursing transfer students. The findings highlight the importance of structured support systems, mentorship, academic advice, and personalized learning pathways to facilitate a positive transition.

## 1. Introduction

Nurses constitute the largest group of healthcare professionals in the South Korean healthcare system [1]. The Korean nursing education system faces distinctive challenges due to the significant gap between the number of licensed and actively practicing nurses [2]. Although South Korea has 19.7 licensed nurses per 1000 people, surpassing the OECD (Organization for Economic Co-operation and Development) average of 13.6. However, only 6.8 nurses per 1000 people are actively working [3]. Addressing this nursing shortage has become a critical policy priority, leading to strategic initiatives to expand the nursing workforce [4]. Expanding transfer opportunities for non-nursing majors into nursing programs was an effective way to grow the nursing workforce [5]. The Enforcement Decree of the Higher Education Act was amended in 2018 to address this demand through collaboration between healthcare stakeholders and educational institutions. As a result of this amendment, the admission quota for nursing transfer students has been provisionally increased to 30% of the total enrollment for five years, enabling up to 4700 students to enroll in nursing programs each year through the transfer pathway [6]. The Accelerated Second-Degree Bachelor of Science in Nurse (ABSN) programs introduced in the US in the 1970s to combat the nurse shortage are comparable to this curriculum [7]. Similar transfer initiatives have been used in other regions, including Europe and Australia, to meet shortages of nurses [8]. While transfer programs promote accelerated entry into the nursing profession, they also give rise to several challenges for students and institutions [9]. Transfer students may encounter challenges in meeting nursing school’s rigorous academic and professional standards in a novel environment [10]. Research indicates that nursing transfer students are twice as likely to drop out of their first year compared to direct-entry students. However, transfer students who complete their course report comparable clinical performance outcomes and high satisfaction with their experience compared to traditional students [11,12].

Generally, transfer students are expected to manage stress, establish new relationships, and adapt rapidly to the academic demands of university life [13]. Integrating into the nursing program may be challenging due to the discrepancy between the needs of nursing education and their prior academic identities [14]. A lack of specialized support networks can intensify isolation during their navigation of a new learning environment [15]. Therefore, peer support programs are important for fostering a sense of belonging and addressing transfer students’ distinctive challenges. Insights derived from their experiences can inform curriculum development and mentorship for their specific needs [16].

Several studies have shown that transfer students experience various challenges, including a lack of time, skepticism about information, devalued prior knowledge [17], a low sense of capability, a lack of academic support from the faculty [18], low confidence [19], feelings of isolation and estrangement [20], and low academic attainment rates [21]. Nursing students’ professional self-esteem and coping strategies positively influence clinical education satisfaction and nursing competency or performance [22]. In contrast, academic burnout and low self-awareness negatively impact clinical practice by increasing stress and reducing adaptation [23].

In the context of Korean nursing education, the collectivism, hierarchical culture, emphasis on theoretical knowledge, and pressure for rapid adaptation create a distinctive learning environment [24]. Previous studies on these students in South Korea have described administrative, educational, and financial challenges [25,26,27,28]. Much of this research has focused on a specific difficulty rather than actively considering their potential strengths, diverse qualities, or dynamic transition process into professional nursing roles.

This study examines nursing transfer students’ difficulties in adapting to the transferring process, how they utilize their strengths to overcome these obstacles, and how they establish their professional identity. The findings will contribute to the development of a structured framework that addresses their unique needs and serves as fundamental evidence for policies addressing the nursing workforce shortage.

## 2. Methods

### 2.1. Study Design

This study explores the lived experiences of nursing transfer students as they adapt to nursing school life. Colaizzi’s method (1978) was used to uncover the essence of their transition through in-depth interviews and analysis. By examining these students’ challenges and coping strategies, this research could suggest improvements for supporting systems in nursing transfer programs.

### 2.2. Participants

Fifteen female nursing transfer students, aged 25 to 32 years (mean: 27.2 years), were recruited from a university in a metropolitan area in South Korea. All participants were fluent in reading and speaking Korean and willing to provide written informed consent. The participants’ academic backgrounds included business, arts, and science. The time since the transfer to the nursing program ranged from 8 to 13 months (mean: 11 months). Students with previous nursing degrees or healthcare work experience were excluded. Recruitment was carried out in collaboration with the nursing department administration. Eligible students were informed about the research during departmental meetings or by email. The researcher made follow-up contact five days after the study details were distributed, and interviews were arranged with those who expressed interest.

### 2.3. Data Collection

Data were collected from 1 July to 31 August 2022 through in-depth semi-structured face-to-face interviews. The purpose of the study was explained during the first meeting, and written informed consent was obtained. Interview durations ranged from 60 to 120 min, depending on the participant’s responses and engagement. The interviews were conducted in private rooms on the university campus to ensure that students felt comfortable expressing their authentic experiences. A nursing faculty member with a doctoral degree conducted all interviews. Both verbal and non-verbal expressions were recorded. Follow-up interviews were conducted after six weeks to clarify any changes in responses. Each interview was conducted using open-ended questions, such as “What is the lived experience of university life as a transfer student?” and “What challenges did you face in adjusting to your new environment in the first year?”. Participants were encouraged to reflect thoughtfully on their experiences, and the researcher used probing questions to elicit further detail where necessary. Data collection was conducted concurrently with analysis. Interviews continued until data saturation was reached, which is the point where no new themes or sub-themes were identified in the interviews. All information was consistently repeated within the last two interviews. All participants confirmed the extracted themes.

Field notes were taken during the interviews to supplement the data and provide contextual information.

### 2.4. Data Analysis

This study followed Colaizzi’s (1978) descriptive phenomenological method, as applied in a previous study [29]. We utilized Colaizzi’s procedural steps as a framework. Two nursing researchers with doctoral degrees conducted the analysis. The process of analysis followed seven stages: (1) reading all individual descriptions of the experience repeatedly; (2) extracting statements considered significant to the participants’ experiences; (3) deriving meanings from significant statements; (4) clustering similar meanings to identify themes; (5) consolidating descriptions that reflect the participants’ experiences; (6) synthesizing the themes of their experiences; (7) integrating themes to capture the fundamental essence of their transferring experiences.

Throughout this process, the researchers used ’bracketing’ as a process intended to examine the researcher’s preconceptions, prior knowledge, and personal biases. This enabled an objective analysis of the participants’ experiences while ensuring that the researcher maintained an open mind to the participant’s perceptions of the phenomenon, avoiding distortion from her interpretive stance. Academic colleagues unrelated to the subject matter peer-reviewed a random sample of the interview transcripts. We ensured quality and credibility by applying Creswell and Poth’s (2016) methods. To improve transparency, we returned transcripts to the participants upon request and sought their confirmation of our interpretations, maintaining orientation to the phenomenon.

### 2.5. Ethical Considerations

This study was approved by the ethics review committee of the Seoul Women’s College of Nursing (IRB No. SWCN-202206-HR-001). Study participants have the right to withdraw their consent at any time. All data were coded and stored securely to maintain anonymity. Data usage was strictly limited to research purposes.

## 3. Results

**Figure 1:**
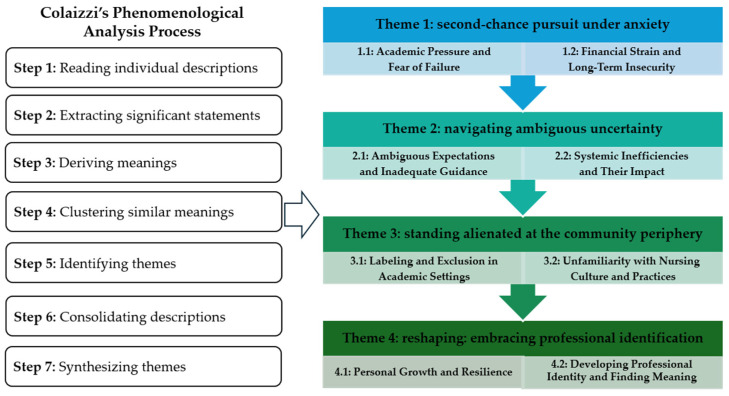
The steps of Colaizzi’s phenomenological analysis and the formation of the main themes of nursing transfer students’ experiences in South Korea.

This study used Colaizzi’s descriptive phenomenological approach to explore the lived experiences of nursing transfer students who are adapting to university life. Data from 15 participants yielded 142 significant statements, which were analyzed and organized into four main themes: (1) second-chance pursuit under anxiety, (2) navigating ambiguous uncertainty, (3) standing alienated at the community periphery, and (4) reshaping: embracing professional identification [Figure 1].

Theme 1: second-chance pursuit under anxiety

Participants described persistent anxiety pervading their daily experiences, emerging from academic, financial, and personal pressures. These concerns created constant tension throughout the transition process.

Many participants expressed feelings of being overwhelmed by the rapid pace of academic demands and the constant pressure to perform well. Fear of failure was particularly pronounced among those who believed their age or prior experience left no room for a second chance.

*“In Korea’s tough job market, age really matters. As an older student, I feel so much pressure to do well academically. I’m older than my classmates. If my grades aren’t good, finding a job will be hard. I can’t afford to mess up, and it makes me anxious. I can’t sleep.”*

*“I thought my STEM background would mean better grades in nursing. But my grades are lower than I expected. It’s frustrating—I need to study more.* *”*

The unfamiliar structure and rigor of nursing education posed challenges that required more time for adaptation.

*“I thought I knew how college systems worked, but clinical trials have been tough … I’m always worried about making mistakes, so I double-check everything.”*

*“Sometimes, I find myself obsessing over small tasks, acting compulsively even for minor things.”*

Economic concerns posed ongoing difficulties, particularly for those juggling prior student loans alongside current tuition fees. These financial pressures compounded their sense of insecurity and anxiety.

*“Tuition again, and I still owe money from my previous course.”*

Participants described their long-term prospects, including doubts about their ability to achieve financial stability or support a family. Starting their careers later heightened these fears, as they needed to “catch up.”

*“Starting later in life makes me want to catch up quickly. Nursing isn’t highly compensated, and I’m worried it won’t be enough to support a family.”*

Most participants reported persistent anxiety as their semester progressed. In their first year, nursing transfer students often experience high levels of anxiety, consistent with previous findings. Financial insecurity has been closely linked to attrition and retention issues in nursing transfer programs [25,26,28].

Theme 2: navigating ambiguous uncertainty

Participants described persistent uncertainty during their adaptation to the nursing program. This uncertainty arose from external factors such as vague expectations, inadequate guidance, and systemic inefficiencies, which complicated their adjustment and heightened anxiety.

*“Professors may think we already know things, but I am not sure what I have learned.”*

*“Nursing has its own language, and it feels like only students who’ve been studying it from the start really get it. So, I don’t know.”*

Participants experienced ambiguity surrounding prerequisites, credit transfers, and rules for nursing programs, which they needed clarification on.

*“The administrative office didn’t provide clear explanations, even for basic requirements like antibody tests.”*

*“If my credits don’t transfer, it delays my graduation and disrupts everything I have planned.”*

*“Hands-on patient care. Right. I don’t know how to be confident with patients or even where to start with bedside manners. It’s overwhelming.”*

Unlike anxiety, which emerged from internal doubts, uncertainty stemmed from external systemic challenges. The clinical practicum exposed the significant gap between theoretical knowledge and practical application in nursing. A low sense of capability and resourcefulness enforced tension, stress, and frustration [8,18]. These discrepancies between the expected support and the actual guidance further contributed to uncertainty and self-doubt among students.

Theme 3: standing alienated at the community periphery

Participants described feeling like outsiders, unable to integrate into their new circumstances. This sense of estrangement emerged from being labeled differently, experiencing exclusion, and struggling with unfamiliar nursing contexts.

*“They always call me a ’transfer student.’ I just want to be treated like others.”*

*“When someone says, ‘We learned this in freshman year,’ it’s like being reminded that I don’t belong yet.”*

Participants often felt excluded from collaborative activities, such as group projects, where they were grouped only with other transfer students.

*“For a group project, all the transfer students were put together. It felt like no one wanted to work with us.”*

*“Can you believe someone on the online community board called us ’free riders’? It was hurtful.”*

*“I didn’t know we needed candles for the pin ceremony or that hand-me-down scrubs were a thing. It just made me feel even more like an outsider.”*

Even in clinical rotation, participants felt their work was undervalued or dismissed as lacking depth.

*“My professor said my clinical rotation report lacked a nursing perspective. It made me wonder if I’ll ever fit in.”*

Participants felt excluded and estranged, not only by traditional students, but also by their own perception. This sense of alienation and stigma led to feelings of being lost and ill-prepared. Despite their diverse backgrounds and strengths, they often considered their previous knowledge to be devalued and faced limited networking opportunities. A lack of belonging within peer groups has been linked to lower academic achievement [20], while strong relationships with non-transferring students play a crucial role in shaping professional nursing identity [17,18,27].

Theme 4: reshaping: embracing professional identification

Despite their struggles, transfer students reshaped themselves through personal growth and professional identity development.

*“I’ve learned to regulate my emotions better when working with my patients. Staying composed has been a major milestone for me.”*

*“I’ve developed greater patience to face difficult situations, even when I felt like giving up.”*

*“I’m discovering ways to solve problems I never imagined I could handle before. It’s like unlocking new potential.”*

Their growth extended to self-awareness and patient-centered care.

*“…Knowing my limits and reaching out for support has helped me feel more balanced.”*

*“My perspective has shifted. Now, I think about what the patients need first.”*

As participants progressed in their nursing education, they developed their professional identities. Clinical experiences served as turning points.

*“Accepting mistakes as part of the learning process has been key to my growth as a nurse.”*

*“In clinical courses, I’m starting to see myself as a future nurse. Each day feels more affirming.”*

Some students found unique importance in their previous experiences.

*“My previous major adds value to how I approach nursing. It gives me perspectives others might not have.”*

*“I keep remembering why I chose this path. The challenges are tough, but they remind me why I’m here as a nurse.”*

Despite multifaceted challenges, these nursing transfer students could develop their professional identity. Supportive communication, self-confidence, self-awareness, and ethical competence were key factors in this process. Prior research indicates that self-efficacy and emotional regulation enhance nursing performance [30], and mentorship strengthens professional identity [26,31]. Establishing a strong professional identity is important for job satisfaction and workforce stability [32].

## 4. Discussion

Securing an adequate supply of nursing students is one of the critical strategies for ensuring future nursing workforce sustainability [33]. South Korea, which faces a persistent nursing shortage, has continuously increased nursing school admission quotas annually. However, simply increasing nursing school admission capacity each year is not a sustainable solution, as demographic shifts—such as declining birth rates—are reducing the potential applicant pool. In response, efforts to diversify the nursing workforce pipeline have included expanding transfer admission pathways into nursing programs. Despite these efforts, nursing transfer students exhibit a notably high attrition rate [34]. Therefore, to secure an adequate and stable nursing workforce, it is crucial to continuously improve transfer student management programs and understand the challenges these students face. This study contributes to this critical need by exploring the lived experiences of nursing transfer students in South Korea.

Anxiety in nursing transfer students reflects pressures across the academic, financial, and personal domains. Participants frequently attributed this anxiety to concerns about their age and perceived limitations in achieving career success, aligning with research on elevated stress in transfer students [35]. Indeed, nursing students often report heightened anxiety during clinical practice due to perceived inadequate preparation [36,37]. Counseling services should be recommended against ageism, collectivism, and a harsh competitive clinical environment; more inclusive and empirical advantages for nursing employment should be provided. Mentorship from successful alumni, particularly those who have navigated career transitions, can provide invaluable emotional and practical support [38]. Prioritizing anxiety mitigation through these comprehensive measures is crucial for improving transfer student well-being, enhancing academic performance, reducing attrition, and fostering a confident and resilient nursing workforce [39,40]. Nursing education must strategically address anxiety to ensure transfer student success and contribute to a sustainable nursing workforce.

Ambiguous expectations and inadequate guidance create uncertainty for nursing transfer students [41,42]. Nursing programs must prioritize clear and proactive communication. This includes transparently outlining program requirements, simplifying administrative processes, and providing detailed clinical practicum information. Skilled clinical educators and advisors can offer personalized guidance, while interactive online resources can address common queries efficiently. Furthermore, the faculty should ensure explicit communication of clinical expectations and provide timely, constructive feedback [18,43]. Reducing ambiguity through these measures can enhance student confidence and program satisfaction, and provide smoother academic and clinical integration [44,45,46]. Therefore, clarity and structured guidance are essential for nursing programs to effectively support transfer students in navigating program uncertainty.

Alienation for nursing transfer students arises from feeling like outsiders, hindering their community integration [47,48]. Participants described labeling, exclusion from peer activities, and devaluation of prior knowledge, impeding belonging and professional identity [20,49]. To alleviate this alienation, nursing programs must actively foster inclusive learning environments. This includes implementing structured peer support programs to connect transfer and traditional students in clinical practicum, promoting group activities for the community that encourage collaboration. Addressing the origins of estrangement will enhance academic achievement and professional identity [17], and cultivate a more diverse and collaborative nursing education environment.

Reshaped professional identification emerged as transfer students demonstrated resilience and transformative growth despite challenges. Participants developed emotional regulation, problem-solving skills, and patient-centered perspectives, showing their adaptive capacity. Nursing education should leverage transfer students’ inherent resilience to foster professional identity formation. This involves strengths-based approaches that value diverse experiences and integrate prior learning into the curriculum, alongside mentorship focused on professional identity development [32,50]. Curriculum integration requires the mapping of prior learning competencies to nursing knowledge. Mentorship programs benefit from clear goals, mentor training, and detailed evaluation. Nurturing resilience and professional identity empowers transfer students, enhances professional commitment, and contributes to a more adaptable, patient-centered nursing workforce. Resilience-focused education is key to students’ transformative potential [21,28].

### Limitations

This study, while offering valuable insights into the participants’ adaptation to nursing school life, is subject to several limitations. Firstly, the timing of data collection may have introduced recall bias. As the interviews were conducted after participants had begun adapting to their new environment, their recollections of initial challenges might have been influenced by subsequent experiences and coping mechanisms. Secondly, the study’s focus on a single metropolitan area limits the transferability of the findings to other regions. Replicating this study in diverse geographical locations, including rural or less urbanized areas, would enhance the findings and provide a broader understanding of adaptation challenges. Thirdly, as the study population consisted exclusively of women, the findings may not be applicable to other demographic groups. Lastly, the absence of perspectives from students who left the program represents a significant limitation. These individuals may have encountered the most significant adaptation challenges, and their insights could provide valuable information for identifying and addressing barriers to successful adaptation. To enhance transparency and rigor, the COREQ (Consolidated Criteria for Reporting Qualitative Research) checklist has been included as Supplementary File S1.

## 5. Conclusions

In conclusion, this study illuminates the unique experiences of South Korean nursing transfer students, revealing critical insights for enhanced support. Moving beyond describing challenges, this research identifies key interventions: implementing personalized counseling protocols with advisors, mandating integration activities, and utilizing reflective portfolio assessments. Intentional, multifaceted support systems are essential for maximizing the potential of these students. In terms of practical applications, nursing programs should prioritize structured mentorship, refined orientation for transfer students, and faculty development for inclusive teaching. Future research should focus on the longitudinal impact of those interventions and explore diverse cultural contexts. Ultimately, evidence-based strategies in nursing education can cultivate thriving transfer students, strengthening the nursing workforce for a more robust healthcare system in South Korea.

## Data Availability

The data supporting this study’s findings were collected through in-depth interviews with the participants. To ensure the confidentiality and privacy of the participants, the data are not publicly available.

## Supplementary Materials

The following supporting information can be downloaded at: https://www.mdpi.com/article/10.3390/nursrep15020072/s1, Supplementary File S1: COREQ checklist. Reference [51] is cited in the supplementary materials.

## Public Involvement Statement

There was no public involvement in any aspect of this research.

## Guidelines and Standards Statement

We have followed the COREQ (Consolidated Criteria for Reporting Qualitative Research) guidelines for qualitative research.

## References

[1] Cheong H.S., Kwon K.T., Hwang S., Kim S.-W., Chang H.-H., Park S.Y., Kim B., Lee S., Park J., Heo S.T. (2022). Workload of Healthcare Workers during the COVID-19 Outbreak in Korea: A Nationwide Survey. J. Korean Med. Sci..

[2] Kim S., Kim S. (2021). The Current Status of the Administrative Dispositions of Nurses: A Nationwide Survey in South Korea. J. Nurs. Res..

[3] Lee J., Lee B. (2022). Psychological Workplace Violence and Health Outcomes in South Korean Nurses. Workplace Health Saf..

[4] Lee N., Lee H.-J. (2020). South Korean Nurses’ Experiences with Patient Care at a COVID-19-Designated Hospital: Growth after the Frontline Battle against an Infectious Disease Pandemic. Int. J. Environ. Res. Public Health.

[5] Lee H.J., Lee N. (2022). Students’ College Life Adaptation Experiences in the Accelerated Second-Degree Bachelor of Science in Nursing Program in South Korea. J. Korean Acad. Soc. Nurs. Educ..

[6] Ministry of Education (2018). Republic of Korea. Partial Amendment to the Higher Education Act; Article 15-2 (Postponement of Acquisition of Bachelor’s Degrees). https://law.go.kr/LSW/eng/engLsSc.do?menuId=2&query=HIGHER%20EDUCATION%20ACT#EJP8:0.

[7] Schwartz J., Gambescia S.F., Patton C. (2017). Impetus and Creation of an Accelerated Second-Degree Baccalaureate Nursing Program Readmission Policy. SAGE Open Nurs..

[8] Saitoh A., Shimoda K., Kawabata A., Oku H., Horiuchi S. (2022). Evaluation of the First Accelerated Bachelor of Science in Nursing Program as a Second Career in Japan. Nurse Educ. Today.

[9] Ma F., Bai Y., Bai Y., Ma W., Yang X., Li J. (2018). Factors Influencing Training Transfer in Nursing Profession: A Qualitative Study. BMC Med. Educ..

[10] Wall P. (2016). Experiences of Nursing Students in a Bachelor of Nursing Program as They Transition from Enrolled Nurse to Registered Nurse. Ph.D. Thesis.

[11] Ching S.S., Zhang L.W., Guan G.Y., Cheung K. (2020). Challenges of University Nursing Transfer Students in an Asian Context: A Qualitative Study. BMJ Open.

[12] Dante A., Ferrão S., Jarosova D., Lancia L., Nascimento C., Notara V., Pokorna A., Rybarova L., Skela-Savič B., Palese A. (2016). Nursing Student Profiles and Occurrence of Early Academic Failure: Findings from an Explorative European Study. Nurse Educ. Today.

[13] Son H.G., Kwon S., Park H.-J. (2017). The Influence of Life Stress, Ego-Resilience, and Spiritual Well-Being on Adaptation to University Life in Nursing Students. J. Korea Acad.-Ind. Coop. Soc..

[14] Cameron C. (2005). Experiences of Transfer Students in a Collaborative Baccalaureate Nursing Program. Community Coll. Rev..

[15] Morosanu L., Handley K., O’Donovan B. (2010). Seeking Support: Researching First-year Students’ Experiences of Coping with Academic Life. High. Educ. Res. Dev..

[16] Lewis C. (2017). Creating Inclusive Campus Communities: The Vital Role of Peer Mentorship in Inclusive Higher Education. Metrop. Univ..

[17] Ralph N., Birks M., Chapman Y., Muldoon N., McPherson C. (2013). From EN to BN to RN: An exploration and analysis of the literature. Contemp. Nurse.

[18] Hutchinson L., Mitchell C., John WS T. (2011). The transition experience of Enrolled Nurses to a Bachelor of Nursing at an Australian university. Contemp. Nurse.

[19] Boelen M., Kenny A. (2009). Supporting enrolled nurse conversion—The impact of a compulsory bridging program. Nurse Educ. Today.

[20] Porter-Wenzlaff L.J., Froman R.D. (2008). Responding to Increasing RN Demand: Diversity and Retention Trends Through an Accelerated LVN-to-BSN Curriculum. J. Nurs. Educ..

[21] Tower M., Cooke M., Watson B., Buys N., Wilson K. (2015). Exploring the Transition Experiences of Students Entering into Preregistration Nursing Degree Programs with Previous Professional Nursing Qualifications: An Integrative Review. J. Clin. Nurs..

[22] Kim K., Lee B. (2014). The Relationship between Satisfaction with Clinical Practice and Clinical Performance Ability for Nursing Students. Int. J. Korea Contents Assoc..

[23] Kim E., Youn Y.J., Lee J. (2019). The Effect of Clinical Practice Transitional Shock and Resilience on Academic Burnout of Nursing Students. Korean Assoc. Learn.-Centered Curric. Instr..

[24] Lee J.J., Yang S.C. (2019). Professional socialisation of nursing students in a collectivist culture: A qualitative study. BMC Med. Educ..

[25] Shin K.R., Cha E.J., Kim Y.H. (2003). The Lived Experience of a Student Transferring into the Nursing Program. J. Korean Acad. Nurs..

[26] Kim M.J., Kim S., Byun E.K. (2016). The Experiences of Students Transferring into the Nursing Program at Local Universities. J. Fish. Mar. Sci. Educ..

[27] Kim J.M., Park K.S., Kim Y.H., Jeong Y.J. (2023). The Experiences of Graduates Who Transferred to a Bachelor of Science in Nursing Program at a College. Korean Soc. Nurs. Res..

[28] Kim I.J., Jeon M.K., Kim Y.S. (2017). Types of Adaptation to College Life for Transfer Nursing Students—Using Q Methodology. Crisisonomy.

[29] Morrow R., Rodriguez A., King N. (2015). Colaizzi’s Descriptive Phenomenological Method. Psychologist.

[30] Yang S. (2015). The Effect of Emotional Intelligence and Self-Efficacy on Clinical Competence of the Nursing Students. Int. J. Korea Contents Assoc..

[31] Vabo G., Slettebø Å., Fossum M. (2022). Nursing students’ professional identity development: An integrative review. Nord. J. Nurs. Res..

[32] Browne C., Wall P., Batt S., Bennett R. (2018). Understanding perceptions of nursing professional identity in students entering an Australian undergraduate nursing degree. Nurse Educ. Pract..

[33] Institute of Medicine (IOM) (2011). The Future of Nursing: Leading Change, Advancing Health.

[34] Ministry of Health and Welfare (2024). Republic of Korea. A Policy Analysis on the Introduction of Accelerated Bachelor of Science in Nursing (ABSN) Program. https://www.mohw.go.kr/board.es?mid=a10411010100&bid=0019.

[35] Mehr K.E., Daltry R. (2016). Examining Mental Health Differences between Transfer and Nontransfer University Students Seeking Counseling Services. J. Coll. Stud. Psychother..

[36] Al-Ghareeb A., McKenna L., Cooper S. (2019). The Influence of Anxiety on Student Nurse Performance in a Simulated Clinical Setting: A Mixed Methods Design. Int. J. Nurs. Stud..

[37] Luo Y., Wang H. (2009). Correlation Research on Psychological Health Impact on Nursing Students against Stress, Coping Way and Social Support. Nurse Educ. Today.

[38] Singer D.L., Sapp A., Baker K.A. (2022). Belongingness in Undergraduate/Pre-Licensure Nursing Students in the Clinical Learning Environment: A Scoping Review. Nurse Educ. Pract..

[39] Bledsoe S., Baskin J.J., Berry F. (2018). Fear Not! How Students Cope with the Fears and Anxieties of College Life. Coll. Teach..

[40] Hutchinson T.L., Janiszewski Goodin H. (2013). Nursing Student Anxiety as a Context for Teaching/Learning. J. Holist. Nurs..

[41] Foster M., Mulroy T., Carver M. (2020). Exploring Coping Strategies of Transfer Students Joining Universities from Colleges. Stud. Success.

[42] Sweeney A.B. (2021). Transitional Challenges in a Two-plus-Two Nursing Program: Phenomenology of Student Experiences. Nurse Educ. Pract..

[43] Fitzgibbon K., Murphy K.D. (2023). Coping Strategies of Healthcare Professional Students for Stress Incurred during Their Studies: A Literature Review. J. Ment. Health.

[44] Charleston R., Happell B. (2005). Coping with Uncertainty within the Preceptorship Experience: The Perceptions of Nursing Students. Psychiatr. Ment. Health Nurs..

[45] Masoudi Alavi N. (2014). Self-Efficacy in Nursing Students. Nurs. Midwifery Stud..

[46] Tantillo M., Marconi M.A., Rideout K., Anson E.A., Reifenstein K.A. (2017). Creating a Nursing Student Center for Academic and Professional Success. J. Nurs. Educ..

[47] Cho H.-K., Chung S.-K. (2015). Relationship among College Life Stress, Alienation and College Adjustment: Focused on Transferred and Non-Transferred Nursing Students. J. Korean Data Anal. Soc..

[48] Lewkonia R. (2001). Educational Implications of Practice Isolation. Med. Educ..

[49] Celerio J.G. (2022). Through the revolving door: Experiences of nursing students. Int. J. Res. Publ..

[50] Cederbaum J., Klusaritz H.A. (2009). Clinical Instruction: Using the Strengths-Based Approach with Nursing Students. J. Nurs. Educ..

[51] Tong A., Sainsbury P., Craig J. (2007). Consolidated criteria for reporting qualitative research (COREQ): A 32-item checklist for interviews and focus groups. Int. J. Qual. Health Care.

