# Supplementary material for: Bridging the Gap: A Phenomenological Study of Transfer Students’ Journey into Professional Nursing
Source: Nurs Rep. 2025 Feb 18;15(2):72. doi: 10.3390/nursrep15020072 (PMC11857965; doi:10.3390/nursrep15020072)
Supplement: Supplementary file 1 [file nursrep-15-00072-s001.zip › nursrep-3406353-supplementary.pdf]

Supplementary File S1: COREQ checklist

Consolidated criteria for reporting qualitative studies (COREQ): 32-item checklist.

Developed from the following:

Tong A, Sainsbury P, Craig J. Consolidated criteria for reporting qualitative research (COREQ): a 32-item checklist for interviews and focus groups. *International Journal for Quality in Health Care*. 2007. Volume 19, Number 6: pp. 349 – 357.

Item No.	Guide Questions/Description	Reported on Page #
Domain 1: research team and reflexivity		
Personal characteristics		
1. Interviewer/facilitator	Which author/s conducted the interview or focus group?	Pg 3
2. Credentials	What were the researcher's credentials, e.g., PhD, MD?	Pg 1
3. Occupation	What was their occupation at the time of the study?	Pg 1
4. Gender	Was the researcher male or female?	Pg 1
5. Experience and training	What experience or training did the researcher have?	Pg 1, 3
Relationship with participants		
6. Relationship established	Was a relationship established prior to study commencement?	Pg 3
7. Participants' knowledge of interviewer	What did the participants know about the researcher, e.g., personal goals, reasons for doing the research?	Pg 3
8. Interviewer characteristics	What characteristics were reported about the interviewer/facilitator, e.g., bias, assumptions, reasons, and interests in the research topic?	Pg 3
Domain 2: study design		
Theoretical framework		

Item No.	Guide Questions/Description	Reported on Page #
9. Methodological orientation and theory	What methodological orientation was stated to underpin the study, e.g., grounded theory, discourse analysis, ethnography, phenomenology, content analysis?	Pg 2,3
Participant selection		
10. Sampling	How were participants selected, e.g., purposive, convenience, consecutive, snowball sampling?	Pg 3
11. Method of approach	How were participants approached, e.g., face-to-face, telephone, mail, email?	Pg 3
12. Sample size	How many participants were in the study?	Pg 1,3
13. Non-participation setting	How many people refused to participate or dropped out? Reasons?	N/A (no drop-out)
14. Setting of data collection	Where were the data collected, e.g., home, clinic, workplace?	Pg 3
15. Presence of nonparticipants	Was anyone else present besides the participants and researchers?	N/A
16. Description of sample	What are the important characteristics of the sample, e.g., demographic data, date?	Pg 3
Data collection		
17. Interview guide	Were questions, prompts, and guides provided by the authors? Was the interview pilot tested?	Pg 3
18. Repeat interviews	Were repeat interviews carried out? If yes, how many?	Pg 3
19. Audio/visual recording	Did the research use audio or visual recording to collect the data?	Pg 3
20. Field notes	Were field notes made during and/or after the interview or focus group?	Pg.3
21. Duration	What was the duration of the interview or focus group?	Pg 5
22. Data saturation	Was data saturation discussed?	Pg 3
23. Transcripts returned	Were transcripts returned to participants for comment and/or correction?	N/A
Domain 3: analysis and findings		
Data analysis		

Item No.	Guide Questions/Description	Reported on Page #
24. Number of data coders	How many data coders coded the data?	Pg 4
25. Description of coding tree	Did the authors provide a description of the coding tree?	N/A
26. Derivation of themes	Were themes identified in advance or derived from the data?	Pg 4,5
27. Software	What software, if applicable, was used to manage the data?	Pg 5
28. Participant checking	Did participants provide feedback on the findings?	Pg 3
Reporting		
29. Quotations presented	Were participant quotations presented to illustrate the themes/findings? Was each quotation identified, e.g., via participant number?	Pg 4-6
30. Consistency of data and findings	Was there consistency between the data presented and the findings?	Pg 4-6
31. Clarity of major themes	Were major themes clearly presented in the findings?	Pg 7
32. Clarity of minor themes	Is there a description of diverse cases or a discussion of minor themes?	Pg 7
